# Types of suicide pacts: a comparative analysis using the National Violent Death Reporting System

**DOI:** 10.3389/fpsyt.2023.1139305

**Published:** 2023-05-05

**Authors:** Kawon Victoria Kim, Cayley Russell, Mark S. Kaplan, Jürgen Rehm, Shannon Lange

**Affiliations:** ^1^Institute for Mental Health Policy Research, Centre for Addiction and Mental Health, Toronto, ON, Canada; ^2^Dalla Lana School of Public Health, University of Toronto, Toronto, ON, Canada; ^3^Ontario Node, Canadian Research Initiative in Substance Misuse (CRISM), Centre for Addiction and Mental Health, Toronto, ON, Canada; ^4^Luskin School of Public Affairs, University of California, Los Angeles, CA, United States; ^5^Campbell Family Mental Health Research Institute, Centre for Addiction and Mental Health, Toronto, ON, Canada; ^6^Department of Psychiatry, University of Toronto, Toronto, ON, Canada; ^7^Institute of Medical Science, University of Toronto, Toronto, ON, Canada; ^8^Institute of Clinical Psychology and Psychotherapy, Technische Universität Dresden, Dresden, Germany; ^9^Department of International Health Projects, Institute for Leadership and Health Management, I.M. Sechenov First Moscow State Medical University, Moscow, Russia; ^10^Zentrum für Interdisziplinäre Suchtforschung (ZIS), Universitätsklinikum Hamburg-Eppendorf, Hamburg, Germany

**Keywords:** suicide, suicide pact, mental health, public health, United States

## Abstract

**Introduction:**

Suicide pacts are lethal acts of violence involving multiple decedents. No study has ever compared suicide pact types using a large sample, limiting our understanding of this rare but serious phenomenon. The objective of the current study was to describe suicide pacts in the United States and empirically compare suicide pacts wherein all decedents died by self-harm with those that involved assisted suicide.

**Methods:**

Using restricted access incident-level data from the National Violent Death Reporting System, we identified 277 suicide pact incidents (225 suicide pacts wherein all decedents died by self-harm and 52 suicide pacts wherein one pact member died by assisted suicide). The two suicide pact types were compared for demographics, pact characteristics, and preceding circumstances.

**Results:**

Compared with decedents of suicide pacts involving assisted suicide, decedents of suicide pacts wherein both members died by self-harm had significantly lower odds of being non-white, Hispanic or non-Hispanic (OR = 0.33, 95%CI: 0.18, 0.64), using an active method of suicide (i.e., ICD-10 codes X70-X83) (OR = 0.01, 95%CI: <0.01, 0.04), and experiencing interpersonal relationship problems (OR = 0.48, 95%CI: 0.27, 0.87) and a crisis within two weeks of their death (OR = 0.58, 95%CI: 0.36, 0.97), but greater odds of preceding physical health problems (OR = 3.25, 95%CI: 1.84, 6.04).

**Discussion:**

Overall, our findings indicate that suicide pacts wherein all decedents died by self-harm and suicide pacts that involved an assisted suicide appear to have largely distinct profiles. While further research is required, the discrete characteristics of these two types of suicide pacts have important implications for prevention.

## 1. Introduction

Suicide pacts, in which two or more people agree to die together, make up a small fraction of suicides (approximately 1% of suicides) (1, 2), but are a persistent and devastating phenomenon in the United States (US). The impact of suicide pacts on family, friends, and society (3) is arguably greater than that of solitary suicides as there are multiple decedents involved. The literature on suicide pacts is largely limited to case reports, except for three studies published in 1961, 1999, and 2009, which used national samples to describe the circumstances and sociodemographic characteristics of suicide pacts in England and Wales (1, 2, 4). Among the existing studies to utilize a national sample, it was found that most suicide pacts involved a passive cause of death (i.e., self-poisoning) and occurred among middle-aged spouses, one or more of whom were facing a fatal or life-altering health condition (1, 2, 4).

Given the dearth of studies in the US, it is unclear whether such characteristics can be generalized to suicide pacts occurring here. Social and cultural differences between countries may result in country-specific profiles of suicide pacts. For instance, it is possible that a greater number of suicide pact decedents die by gunshot wounds in the US, where there is greater accessibility to guns, than in a country with stricter gun control policies. Further, while existing studies have compared suicide pacts to solitary suicides and have found that suicide pact decedents are more likely female, older and employ passive methods of death (e.g., carbon monoxide poisoning) compared to solitary suicide decedents (4), they have failed to differentiate between different types of pacts. To date, no study has compared suicide pacts wherein both decedents died by self-harm with suicide pacts wherein one member of the suicide pact kills another pact member, with the permission of that pact member, and then dies by self-harm. This oversight is likely due to the rarity of suicide pacts involving assisted suicide and the difficulty of ascertaining such cases (due to an objective element, such as a suicide note and motive, being required to prove that an incident is a suicide pact), and therefore, the scarceness of data as a result.

The current study intends to fill the literature gaps identified above. As such, the objective was to describe, for the first time, suicide pacts in the US and empirically compare suicide pacts wherein all decedents died by self-harm with suicide pacts that involved assisted suicide.

## 2. Materials and methods

### 2.1. Data source

Restricted access data from the National Violent Death Reporting System (NVDRS)–a state-based active surveillance system in the United States that provides a detailed account of violent deaths in participating states–for 2003–2019 were used. The NVDRS restricted access data is a de-identified, multistate, incident-level data set, comprising of hundreds of unique variables, obtained from numerous sources, including coroners and medical examiners’ reports, toxicology reports, death certificates, law enforcement records, and supplementary homicide reports (5). The database includes short narratives taken from coroner/medical examiner and law enforcement reports to describe the circumstances of the incident. Although a relatively small number of states participated in the NVDRS in 2003, data were available for 44 states by 2019 (Supplementary Material S1).

### 2.2. Measures

For the current study, a suicide pact was defined as “a mutual arrangement between two [or more] people who resolve to die at the same time and, nearly always, in the same place” (2). If one of the decedents of a suicide pact died by assisted suicide (i.e., assisted by the other pact member), these incidents were referred to as a suicide pact involving assisted suicide. The systematic inclusion and exclusion criteria used to identify and categorize suicide pacts are presented in Supplementary Material S2.

Demographic factors included age, sex, and race/ethnicity. Additional variables included cause of death, the relationship between pact members and the circumstances preceding the incident. Per the NVDRS data sharing agreement, cells showing or derived from fewer than 10 deaths must be suppressed. Therefore, in such cases, categories were collapsed (e.g., dichotomized)–this was the case for race/ethnicity (dichotomized as non-white, Hispanic or non-Hispanic; or white, non-Hispanic), and cause of death (dichotomized as passive (intentional self-poisoning, ICD-10 codes: X60-X69) or active (all other methods, ICD-10 codes: X70-X83) methods). Suicide incidents that coded as intentional self-harm by unspecified means (ICD-10 code: X84) were excluded from the respective analyses. If an incident did not have a suicide-specific ICD-10 code as the cause of death, the coroner/medical examiner and law enforcement narratives, and information available for the variables cause of death and weapon type, were reviewed to ascertain the method of suicide. Those without enough information to determine if the method was active or passive were excluded from the respective analyses.

Given that the NVDRS does not provide a variable to describe the relationship between pact members, the narratives taken from the coroner/medical examiner and law enforcement report were reviewed to ascertain their relationship, and then were subsequently dichotomized as either romantic partners or family/friends/close acquaintances. Preceding circumstances, defined as an event occurring within an undefined period of time that appears to have contributed to the death, included death of a family member or friend (including suicide), depressed mood, eviction or loss of home, financial problem(s), interpersonal relationship problem(s), involvement with the legal system, job and/or school problem(s), physical health problem(s), and any crisis within two weeks of the incident (5). The NVDRS defines a “crisis” as any of the former preceding circumstances, and mental health or substance use crises, if they occurred within 14 days of their death. If a preceding circumstance or crisis was endorsed, this means that the coroner/medical examiner or law enforcement officials felt the respective problem or event had contributed to the death of the decedent.

### 2.3. Statistical analysis

The demographics, pact characteristics, and preceding circumstances of suicide pacts involving an assisted suicide were compared with suicide pacts wherein both decedents died by self-harm. Chi-square tests were used for the descriptive analysis of categorical variables. For age (a continuous variable), Welch’s two-sample *t*-test for normally distributed data was used. Two proportion z-tests were utilized to determine if the proportions were significantly different for each preceding circumstance category. Odds ratios (OR) with 95% confidence intervals (CI) were calculated for categorical variables and were estimated using conditional maximum likelihood estimation. If decedents were missing information for a variable, they were excluded from the total count (*n*) when calculating proportions and running statistical tests. All analyses were performed using RStudio version 1.3.1073 (6).

## 3. Results

A total of 225 suicide pacts wherein all decedents died by self-harm, with ages ranging from 14 to 98 years old (mean age = 57.13, SD = 21.67), and 52 suicide pacts that involved assisted suicide, with ages ranging from 15 to 86 years old (mean age = 55.13, SD = 19.56), were identified in the NVDRS (Table 1). The two suicide pact types did not differ significantly in age or relationship type between pact members. However, decedents of suicide pacts wherein all members died by self-harm had lower odds (OR = 0.33, 95% CI: 0.18, 0.64) of being non-white, Hispanic or non-Hispanic, and lower odds (OR = 0.01, 95% CI: <0.01, 0.04) of using an active method of suicide, compared with decedents of suicide pacts wherein one member died by assisted suicide (Table 2).

**Table 1:** Socio-demographics, pact characteristics, and preceding circumstances of suicide pacts in the United States, by pact type.

	Suicide pacts (All)	Suicide pacts wherein all decedents died by self-harm	Suicide pacts that involved an assisted suicide	Test statistic	*p*-value
Number of incidents	277	225	52		
Number of individuals	554	450	104		
Mean age (SD)^d^	56.76 (21.29)	57.13 (21.67)	55.13 (19.56)	−0.916	0.361
Sex, n (%)^d^				0.240	0.625
Male	273 (49.3)	224 (49.8)	49 (47.1)		
Female	281 (50.7)	226 (50.2)	55 (52.9)		
Race/ethnicity, *n* (%)^d^				14.328	**<0.001**
Non-white, Hispanic or non-Hispanic	56 (10.1)	35 (7.8)	21 (20.2)		
White, non-Hispanic	498 (89.9)	415 (92.2)	83 (79.8)		
Cause of death (method), *n* (%)^b^^,^^d^				110.100	**<0.001**
Passive (X60-X69)	263 (47.7)	262 (58.6)	<10^a^		
Active (X70-X83)	288 (52.3)	185 (41.4)	>90^a^		
Relationship between pact members, *n* (%)^c^^,^^e^					
Romantic partners	203 (81.5)	162 (81.4)	>40^a^	0.009	0.923
Family/friends/close acquaintances	46 (18.5)	37 (18.6)	<10^a^		
Circumstances preceding the incident, *n* (%)^d^					
Death of a family member or friend, including suicide	46 (8.3)	34 (7.6)	12 (11.5)		0.185
Depressed mood	79 (14.3)	70 (15.6)	<10^a^		0.070
Eviction or loss of home	23 (4.2)	17 (3.8)	<10^a^		0.359
Financial problem(s)	70 (12.6)	60 (13.3)	10 (9.6)		0.304
Interpersonal relationship problem(s)	77 (13.9)	54 (12.0)	23 (22.1)		**0.007**
Involvement with the legal system	53 (9.6)	45 (10.0)	<10^a^		0.471
Job and/or school problem(s)	22 (4.0)	20 (4.4)	<10^a^		0.364
Physical health problem(s)	192 (34.7)	175 (38.9)	17 (16.3)		**<0.001**
Any crisis within 2 weeks of the incident	129 (23.3)	96 (21.3)	33 (31.7)		**0.024**

a As per the NVDRS, cells showing or derived from fewer than 10 deaths must be suppressed; as such, in such instances the n is not reported and the proportion is presented as either <10 or > *n*–10.

b Excludes undetermined causes of death (ICD-10 code X84: Intentional self-harm by unspecified means).

c Excludes incidents for which the relationship between pact members was unknown.

d Reported at the individual level.

e Reported at the suicide pact incident level. Bold values denote statistical significant at the *p*<0.05 level.

Experiencing physical health problems, interpersonal relationship problems, and any crisis within two weeks of the incident were the only preceding circumstances that produced a statistically significant OR (Table 2). While decedents of suicide pacts wherein all members died by self-harm had greater odds of experiencing preceding physical health problems (OR = 3.25, 95% CI: 1.84, 6.04) in comparison to decedents of suicide pacts that involved an assisted suicide, they had lower odds of experiencing interpersonal relationship problems (OR = 0.48, 95% CI: 0.27, 0.87) and a crisis (OR = 0.58, 95% CI: 0.36, 0.97).

**Table 2:** Odds ratios for demographics, pact characteristics, and preceding circumstances for suicide pacts wherein all decedents died by self-harm, compared with suicide pacts that involved an assisted suicide.

Variable		OR	95% CI
Demographics and pact characteristics			
Sex (ref: Male)	Female	0.90	0.57, 1.41
Race/ethnicity (ref: White, non-Hispanic)	Non-white, Hispanic or non-Hispanic	**0.33**	**0.18, 0.64**
Cause of death (method) (ref: Passive)	Active	**0.01**	**<0.01,0.04**
Relationship between pact members (ref: Romantic partner)	Family/friend/close acquaintance	1.04	0.58, 1.96
Preceding circumstances^a^			
Death of a family member or friend, including suicide	Yes	0.63	0.30, 1.38
Depressed mood	Yes	1.94	0.92, 4.59
Eviction or loss of home	Yes	0.64	0.23, 2.04
Financial problem(s)	Yes	1.45	0.70, 3.29
Interpersonal relationship problem(s)	Yes	**0.48**	**0.27, 0.87**
Involvement with the legal system	Yes	1.33	0.60, 3.38
Job and/or school problem(s)	Yes	2.37	0.56, 21.23
Physical health problem(s)	Yes	**3.25**	**1.84, 6.04**
Any crisis within 2 weeks of the incident	Yes	**0.58**	**0.36, 0.97**

ref, reference category.

a Presents odds ratios computed with “No, not available, unknown” as the reference category. Bold values denote statistical significant at the *p*<0.05 level.

## 4. Discussion

In this brief report, we have, for the first time, described a large sample of suicide pacts in the US and empirically compared two types of suicide pacts. Although there are similarities, suicide pacts wherein both decedents died by self-harm had distinct characteristics from suicide pacts that involved assisted suicide, particularly with respect to race/ethnicity, the cause of death, and certain preceding circumstances (i.e., interpersonal relationship problems, physical health problems, and a crisis within two weeks of the incident). The differentiation of the two types of suicide pacts investigated here suggests that targeted interventions may differ for each. The finding that suicide pacts wherein both decedents died by self-harm were significantly less likely to die by an active cause of death than pacts involving assisted suicide is understandable as self-poisonings (i.e., passive methods) are by and large self-inflicted and do not necessitate assistance. However, future research should consider comparing suicide pacts involving assisted suicide with incidents of homicide followed by suicide, where the suicide decedent was the perpetrator, as such incidents involve active methods more often than not (7, 8). Also, the high proportion of active methods used in suicide pacts wherein one pact member died by assisted suicide highlights that ‘means restriction’ may be particularly impactful in preventing such suicide pacts–e.g., installation of barriers at jump sites or stricter regulation of firearms.

Among the preceding circumstances which were investigated, a statistically significant association was found for experiencing physical health problems, interpersonal relationship problems, or a crisis (i.e., an event occurring within two weeks of the incident that was thought to have contributed to the death of the decedent). It is likely that individuals affected by such circumstances would consequently interact with primary health care or emergency services, thereby identifying a possible point of intervention for suicide pact prevention efforts. Furthermore, all suicide pacts were carried out in pairs of individuals with close relationships, such as spouses, romantic partners, family, friends or close acquaintances. Existing studies and case series on suicide pacts have consistently described older married couples who agree to die by suicide together (5, 9), typically because one of the individuals is suffering from a fatal or life-altering health condition (1, 2, 10), and adolescent friends and romantic partners who enter a suicide pact as a result of loneliness, disapproved relationships, or parental problems (1, 9). Therefore, threatened relationships between two particularly close individuals may be a significant driver of suicide pacts.

### 4.1. Limitations

While the majority of US states had participated in providing data to the NVDRS by 2019, the data are not necessarily nationally representative. Also, abstractors are limited to the information they receive in the incident reports. This is particularly relevant to the preceding circumstances variables. In the NVDRS, preceding circumstances are binary variables for which either “yes” or “no, not available, or unknown” can be endorsed. Therefore, although the former category likely represents a true presence, the latter may not represent a true absence of a particular circumstance.

## 5. Conclusion

In conclusion, suicide pacts wherein all decedents died by self-harm and suicide pacts that involved assisted suicide appear to have distinct profiles, particularly with respect to the preceding circumstances. This study not only draws attention to the occurrence of suicide pacts and the two different types, but also to the distinct characteristics that may increase the risk of dying in a suicide pact. Thus, as discussed above, this study has important implications for suicide prevention efforts and should be viewed as a first step in achieving a greater understanding of this rare but serious phenomenon in the US. Once the sample of suicide pact incidents increases in the NVDRS, more advanced statistical methods to explore the profiles of suicide pacts (e.g., regression analyses or latent class analyses) should be considered.

## Data availability statement

The original contributions presented in the study are included in the article/Supplementary material, further inquiries can be directed to the corresponding author.

## Ethics statement

The studies involving human participants were reviewed and approved by Centre for Addiction and Mental Health, Research Ethics Board (REB# 096/2022). Written informed consent from the participants’ legal guardian/next of kin was not required to participate in this study in accordance with the national legislation and the institutional requirements.

## Author contributions

KK prepared, analysed and interpreted the data, and prepared the first draft of the manuscript. CR contributed to data interpretation and revised the paper. SL led the conception and design of the study, contributed to the statistical analysis, interpretation of the data, and writing of the paper. JR and MK reviewed the manuscript for important intellectual content. All authors contributed to the article and approved the submitted version.

## Conflict of interest

The authors declare that the research was conducted in the absence of any commercial or financial relationships that could be construed as a potential conflict of interest.

## Publisher’s note

All claims expressed in this article are solely those of the authors and do not necessarily represent those of their affiliated organizations, or those of the publisher, the editors and the reviewers. Any product that may be evaluated in this article, or claim that may be made by its manufacturer, is not guaranteed or endorsed by the publisher.

## Supplementary material

The Supplementary material for this article can be found online at: https://www.frontiersin.org/articles/10.3389/fpsyt.2023.1139305/full#supplementary-material
