# Supplementary material for: Types of suicide pacts: a comparative analysis using the National Violent Death Reporting System
Source: Front Psychiatry. 2023 May 5;14:1139305. doi: 10.3389/fpsyt.2023.1139305 (PMC10196347; doi:10.3389/fpsyt.2023.1139305)
Supplement: Supplementary file 1 [file Table_1.docx]

Supplementary Material 1

Types of suicide pacts: A comparative analysis using the National Violent Death Reporting System

Kawon Victoria Kim^1,2^, Cayley Russell^1,3^, Mark S. Kaplan^4^, Jürgen Rehm^1-3,5-10^, and Shannon Lange^1,5,6^*

*** Correspondence:** Shannon Lange, shannon.lange@camh.ca

# NVDRS participating states by year, which were included in the current study

| State | 2003 | 2004 | 2005 | 2006 | 2007 | 2008 | 2009 | 2010 | 2011 | 2012 | 2013 | 2014 | 2015 | 2016 | 2017 | 2018 | 2019 |
| --- | --- | --- | --- | --- | --- | --- | --- | --- | --- | --- | --- | --- | --- | --- | --- | --- | --- |
| Alabama |  |  |  |  |  |  |  |  |  |  |  |  |  |  |  | X | X |
| Alaska | X | X | X | X | X | X | X | X | X | X | X | X | X | X | X | X | X |
| Arizona |  |  |  |  |  |  |  |  |  |  |  |  | X | X | X | X | X |
| California |  |  |  |  |  |  |  |  |  |  |  |  |  |  | X^a^ | X^b^ | X^c^ |
| Colorado |  | X | X | X | X | X | X | X | X | X | X | X | X | X | X | X | X |
| Connecticut |  |  |  |  |  |  |  |  |  |  |  |  | X | X | X | X | X |
| Delaware |  |  |  |  |  |  |  |  |  |  |  |  |  |  | X | X | X |
| District of Columbia |  |  |  |  |  |  |  |  |  |  |  |  |  |  | X | X | X |
| Georgia |  | X | X | X | X | X | X | X | X | X | X | X | X | X | X | X | X |
| Hawaii |  |  |  |  |  |  |  |  |  |  |  |  | X | X |  |  | X |
| Illinois |  |  |  |  |  |  |  |  |  |  |  |  |  | X^d^ | X^d^ | X^d^ | X^d^ |
| Indiana |  |  |  |  |  |  |  |  |  |  |  |  |  | X | X | X | X |
| Iowa |  |  |  |  |  |  |  |  |  |  |  |  |  | X | X | X | X |
| Kansas |  |  |  |  |  |  |  |  |  |  |  |  | X | X | X | X | X |
| Kentucky |  |  | X | X | X | X | X | X | X | X | X | X | X | X | X | X | X |
| Louisiana |  |  |  |  |  |  |  |  |  |  |  |  |  |  |  | X | X |
| Maine |  |  |  |  |  |  |  |  |  |  |  |  | X | X | X | X | X |
| Maryland | X | X | X | X | X | X | X | X | X | X | X | X | X | X | X | X | X |
| Massachusetts | X | X | X | X | X | X | X | X | X | X | X | X | X | X | X | X | X |
| Michigan |  |  |  |  |  |  |  |  |  |  |  | X | X | X | X | X | X |
| Minnesota |  |  |  |  |  |  |  |  |  |  |  |  | X | X | X | X | X |
| Missouri |  |  |  |  |  |  |  |  |  |  |  |  |  |  |  | X | X |
| Montana |  |  |  |  |  |  |  |  |  |  |  |  |  |  |  |  | X |
| Nebraska |  |  |  |  |  |  |  |  |  |  |  |  |  |  |  | X | X |
| Nevada |  |  |  |  |  |  |  |  |  |  |  |  |  |  | X | X | X |
| New Hampshire |  |  |  |  |  |  |  |  |  |  |  |  | X | X | X | X | X |
| New Jersey | X | X | X | X | X | X | X | X | X | X | X | X | X | X | X | X | X |
| New Mexico |  |  | X | X | X | X | X | X | X | X | X | X | X | X | X | X | X |
| New York |  |  |  |  |  |  |  |  |  |  |  |  | X | X | X | X |  |
| North Carolina |  | X | X | X | X | X | X | X | X | X | X | X | X | X | X | X | X |
| North Dakota |  |  |  |  |  |  |  |  |  |  |  |  |  |  |  |  | X |
| Ohio |  |  |  |  |  |  |  |  | X | X | X | X | X | X | X | X | X |
| Oklahoma |  | X | X | X | X | X | X | X | X | X | X | X | X | X | X | X | X |
| Oregon | X | X | X | X | X | X | X | X | X | X | X | X | X | X | X | X | X |
| Pennsylvania |  |  |  |  |  |  |  |  |  |  |  |  |  | X^d^ | X^d^ | X^d^ | X^d^ |
| Puerto Rico |  |  |  |  |  |  |  |  |  |  |  |  |  |  | X | X | X |
| Rhode Island |  | X | X | X | X | X | X | X | X | X | X | X | X | X | X | X | X |
| South Carolina | X | X | X | X | X | X | X | X | X | X | X | X | X | X | X | X | X |
| Utah |  |  | X | X | X | X | X | X | X | X | X | X | X | X | X | X | X |
| Vermont |  |  |  |  |  |  |  |  |  |  |  |  | X | X | X | X | X |
| Virginia | X | X | X | X | X | X | X | X | X | X | X | X | X | X | X | X | X |
| Washington |  |  |  |  |  |  |  |  |  |  |  |  |  | X^d^ | X^d^ | X | X |
| West Virginia |  |  |  |  |  |  |  |  |  |  |  |  |  |  | X | X | X |
| Wisconsin |  | X | X | X | X | X | X | X | X | X | X | X | X | X | X | X | X |
| Wyoming |  |  |  |  |  |  |  |  |  |  |  |  |  |  |  |  | X |
| Total | 7 | 13 | 16 | 16 | 16 | 16 | 16 | 16 | 17 | 17 | 17 | 18 | 27 | 32 | 37 | 41 | 44 |

^a^ Collected data for violent deaths that occurred in 4 counties (representing 27.8% of violent deaths that occurred in California in 2017), in accordance with requirements under which the state was funded.

^b^ Collected data for violent deaths that occurred in 21 counties (representing 55.1% of violent deaths that occurred in California in 2018), in accordance with requirements under which the state was funded.

^c^ Collected data for violent deaths that occurred in 30 counties (representing 55.3% of violent deaths that occurred in California in 2019), in accordance with requirements under which the state was funded.

^d^ Collected data on >80% of violent deaths in state, in accordance with requirements under which the state was funded.
