# Supplementary material for: Types of suicide pacts: a comparative analysis using the National Violent Death Reporting System
Source: Front Psychiatry. 2023 May 5;14:1139305. doi: 10.3389/fpsyt.2023.1139305 (PMC10196347; doi:10.3389/fpsyt.2023.1139305)
Supplement: Supplementary file 2 [file Data_Sheet_1.docx]

Supplementary Material 2

Types of suicide pacts: A comparative analysis using the National Violent Death Reporting System

Kawon Victoria Kim^1,2^, Cayley Russell^1,3^, Mark S. Kaplan^4^, Jürgen Rehm^1-3,5-10^, and Shannon Lange^1,5,6^*

*** Correspondence:** Shannon Lange: shannon.lange@camh.ca

# Suicide Pact Eligibility Criteria

The following step-wise procedure was employed to identify incidents of suicide pacts in the National Violent Death Reporting System (NVDRS):

- Using the variable “IncidentCategory_c”, any incidents labelled as a single suicide in the NVDRS were excluded.
- Using the variables “NarrativeCME”, “NarrativeLE”, “CME_CircumstancesOtherText”, and “LE_CircumstancesOtherText”, any incidents that were explicitly described as being part of a suicide pact were included.
- For incidents involving two or more victims that were not explicitly identified in the NVDRS as being part of a suicide pact, but for which at least one decedent had died by suicide, the narratives from the coroner/medical examiner and law enforcement (variables specified above) were reviewed and included if the narratives explicitly stated, clearly implied, or provided sufficient information to infer that there was an agreement between the decedents to die together (e.g., the presence of a co-signed or individually written suicide note from each member of the suicide pact, or no evidence of foul play).
- Incidents where the manner of death for a decedent was not self-inflicted (i.e., the manner of death was classified as a homicide or the case was described as a “mercy killing”), the case was included for analysis only if the narratives explicitly stated, clearly implied, or provided sufficient information to infer that the decedent’s death was consensual.
- For incidents where the manner of death of one or more decedent could not be determined as either suicide or assisted suicide, all cases linked to the incident were excluded.
- Finally, suicide pact incidents that occurred among adults but involved collateral deaths of children were excluded. In such incidents, it could not be determined with certainty that the children had consented to participate in the suicide pact and therefore did not meet our definition of a suicide pact.
